# Prevalence of Prenatal Depression Among Pregnant Women and the Importance of Resilience: A Multi-Site Questionnaire-Based Survey in Mainland China

**DOI:** 10.3389/fpsyt.2020.00374

**Published:** 2020-05-06

**Authors:** Lijuan Zhang, Xiaoshi Yang, Jinfeng Zhao, Weiyu Zhang, Can Cui, Fengzhi Yang, Ruqing Ma, Yajing Jia

**Affiliations:** ^1^Department of Obstetrics and Gynecology, Shengjing Hospital of China Medical University, Shenyang, China; ^2^Department of Social Medicine, School of Public Health, China Medical University, Shenyang, China; ^3^Faculty of Medical and Health Sciences, The University of Auckland, Auckland, New Zealand

**Keywords:** prenatal depression, perceived stress, resilience, worries about appearance, harmonious relationship

## Abstract

**Background:**

Prenatal depression is associated with adverse maternal and fetal health consequences, yet it has not raised sufficient concerns in China. Psychological worries and stress may lead to prenatal depression, but resilience could relieve some of the negative effects of worries and stress and mitigate prenatal depression.

**Aims:**

This study aimed to assess the prevalence of prenatal depression and explore its associated factors.

**Method:**

A multisite cross-sectional study was conducted that included 605 pregnant women from three hospitals in two provincial capitals (Shenyang and Zhengzhou) and one municipality (Chongqing). A smartphone questionnaire was employed to assess prenatal depression using the Center for Epidemiologic Studies Depression Scale (CES-D). Multivariate logistic regression analysis was performed to explore factors associated with prenatal depression.

**Results:**

The prevalence of prenatal depression (CES-D≥16) among Chinese pregnant women was 28.4%. Logistic regression analyses revealed that prenatal depression was positively associated with worries about appearance (odds ratio [OR] 1.666, 95% confidence interval [CI] 1.043–2.661) and perceived stress (OR 1.156, 95% CI 1.104–1.211) and negatively associated with monthly income, relationship with mother (OR 0.287, 95% CI 0.103–0.796), and resilience (OR 0.935, 95% CI 0.918–0.953).

**Conclusion:**

These findings revealed that Chinese pregnant women suffered from high levels of prenatal depression (28.4%). Worries about appearance and perceived stress were risk factors for prenatal depression, whereas a pregnant woman’s harmonious relationship with her own mother and resilience could relieve the negative impacts of pregnancy that can lead to prenatal depression. Improving resilience and maintaining harmonious relationships with mothers should be emphasized in order to reduce the detrimental effects of pregnancy and improve the mental well-being of pregnant women.

## Introduction

Most pregnant women experience emotional worries and stress during pregnancy: new responsibilities related to their lives and family expectations may lead to depression in both prenatal and postnatal periods (1). Specifically, substantial changes in body function and appearance and worries about fetal health pose great challenges to psychological health, which can result in mental disorders such as depression and anxiety (2, 3).

Prenatal depression is one of the most widespread mental disorders among women during pregnancy, with estimated rates of depression ranging from 7 to 37.1% (4–6). Previous studies indicated that prenatal depression was a strong predictor of postnatal depression, but prenatal depression is more prevalent than postnatal depression (7). In China, there is a scarcity of mental healthcare for women, especially for pregnant women, which can lead not only to adverse health outcomes for the pregnant women themselves but also result in detrimental health outcomes for fetuses and infants, such as abortion, premature birth, low birth weight, and delayed growth and development (8, 9). However, few studies have explored prenatal depression in women in the middle and late stages of pregnancy in China. A previous study found that high resilience can maintain the hypothalamus-pituitary-adrenal axis at an optimal level of activation, making it easier for resilient individuals to avoid psychosomatic disorders such as depression and anxiety (10). According to the social-ecological model, variables predicting the prevalence of prenatal depression can be categorized into individual factors (11) (such as demographic characteristics, pregnancy status, and worries and stress surrounding pregnancy), interpersonal factors (such as relationships with family members and emotional support), and coping and appraisal skills (such as positive psychological abilities, like resilience, that have the potential to enable women to resume normal functioning and engage in activities that help them respond effectively to the process of adaptation). It has been well documented that stress is highly associated with depression (12) among pregnant women. Previous studies have also revealed that worries resulting from pregnancy have emerged as factors that are strongly associated with mental health (13). Worries about fetal sex, appearance changes, and the negative impacts of pregnancy on daily life may have detrimental effects on the prevalence of prenatal depression. Women are susceptible to adverse effects of pregnancy, and, as a result, they experience both physical and psychological changes and negative effects on their daily lives, resulting in an increased risk of depression. Conversely, interpersonal factors, including emotional support and harmonious relationships with family members such as husbands, mothers, and mothers-in-law can positively affect the mental health of pregnant women (14, 15).

Depression can be regulated by positive psychological abilities like resilience. Resilience, as a personality trait and a positive psychological capability, can help individuals adapt to stressful environments or recover after facing difficulties (16). Women with high levels of resilience are flexible and can manage stress rationally to achieve positive psychological outcomes when faced with adversity. A growing number of studies have indicated that resilience acts as a protective factor for mental health and overall positive well-being and functioning in society (17, 18).

**Figure 1:**
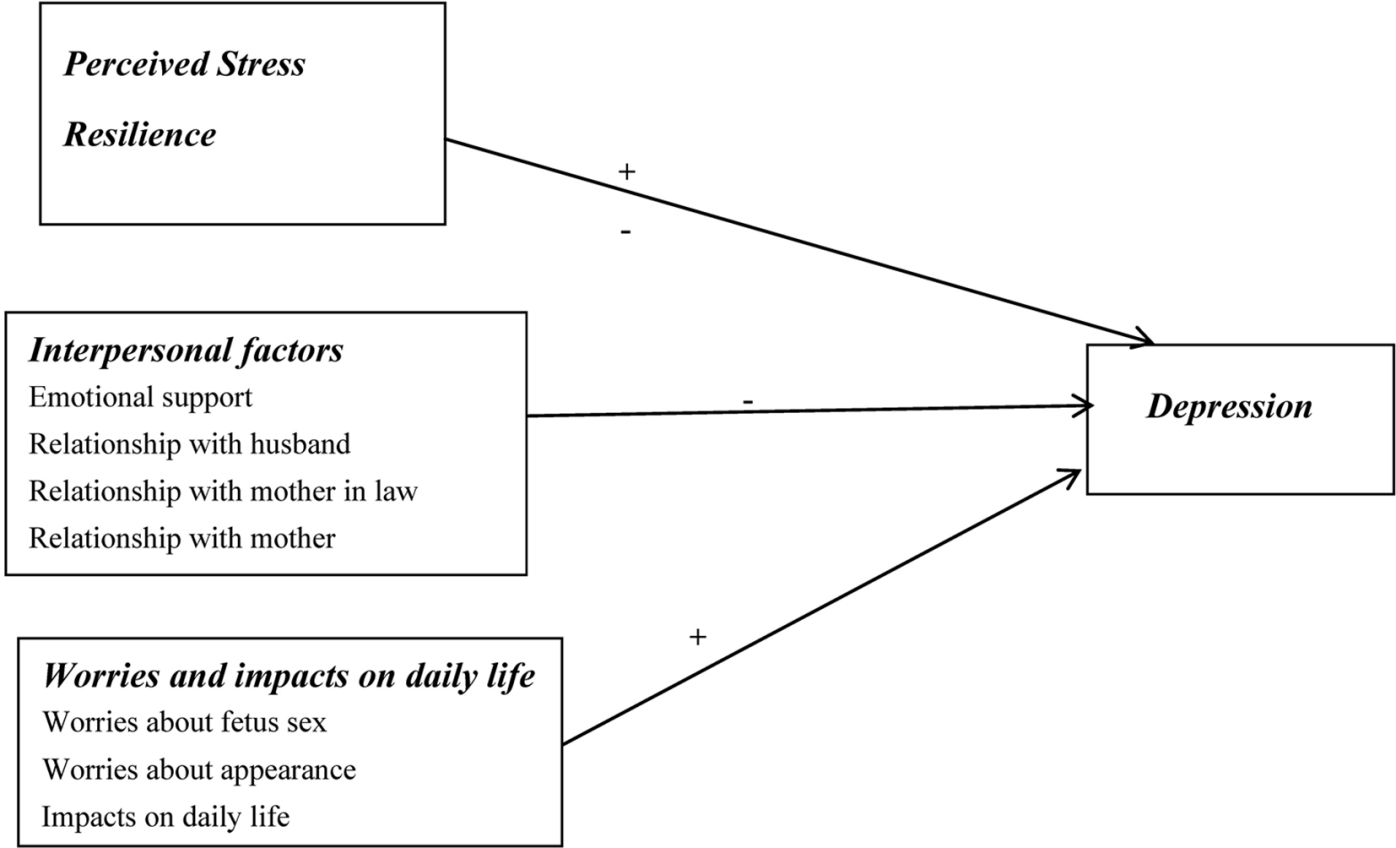
Theoretical framework of prenatal depression in pregnant women. +:Positive correlation; -: Negative correlation.

Research on prenatal mental disorders has only been conducted in a few regions in mainland China (19–21), including Shanghai and Chongqing. Multisite studies focusing on prenatal depression are relatively rare, especially for women in the second and third trimesters of pregnancy. Thus, this study aimed to evaluate the prevalence of prenatal depression experienced by Chinese pregnant women and explore its associated factors. The underlying hypothesis was that perceived stress and impacts on daily life, such as worries about fetal sex, worries about appearance, and changes to daily routines and relationships, would be positively associated with prenatal depression, but resilience and interpersonal factors, such as emotional support and harmonious relationships with husbands, mothers, and mothers-in-law, would be negatively associated with prenatal depression. The theoretical framework is outlined in **Figure 1**.

## Materials And Methods

### Study Design and Sample

A multisite smartphone questionnaire-based cross-sectional study with systematic sampling was performed between July 2018 and July 2019 in three cities (two provincial capital cities [Shenyang in Liaoning Province and Zhengzhou in Henan Province] and one municipality [Chongqing]). This study was conducted in accordance with and approved by the ethical standards of the Committee on Human Experimentation of China Medical University (CMU2018-033).

A total of 800 Chinese pregnant women in the middle and late stages of pregnancy seeking prenatal care who underwent routine prenatal examinations at the obstetrics clinics in tertiary hospitals (including the Second Affiliated Hospital of China Medical University, Zhengzhou Maternal and Child Health Hospital, and Chongqing Bishan District People’s Hospital) were recruited as participants. About 30% of the total pregnant women who underwent routine prenatal examinations at the obstetrics clinics in each hospital were randomly selected according to the outpatients clinic visit numbers. The inclusion criteria were: i) pregnant women aged 18 years or older seeking prenatal care who underwent routine prenatal examinations at the obstetrics clinics; ii) women who were legally married in mainland China; iii) confirmed gestation of 12 weeks or longer; and iv) women who could understand the content of the mobile phone questionnaires. The exclusion criteria were i) women with serious mental disorders or acute infectious diseases; ii) illicit drug use; iii) abnormal pregnancies such as fetal malformations or stillbirths; and iv) women who desired to have an abortion.

Pregnant women who participated in this study were asked to sign the online consent forms and were then enrolled in the study. The participants completed the online questionnaires using a mobile phone *via* Chinese WENJUANXING platform, and a trained surveyor of the research team was present to address questions and explain the purpose of this survey. Of the total 800 women identified, 195 declined to participate; the remaining 605 pregnant women took part and responded effectively in the study, resulting in a 75.63% effective consent rate. The characteristics (age, chronic disease status, and weeks of gestation) of the 605 pregnant women included in the analysis were similar to those of the 195 women who were excluded.

### Measurements

#### Demographic Characteristics of Pregnant Women

Demographic characteristics of the pregnant women, including age, educational level (junior college and below, bachelor’s degree and above), monthly income (≤3,000 yuan, 3,001–5,000 yuan, > 5,000 yuan), weeks of gestation, and the presence of chronic diseases, were collected. Chronic disease was assessed through the question: “Are you suffering from any of the following diseases: cancer, cardiovascular disease, diabetes, arthritis, chronic kidney disease, or stomach disease?”, to which a respondent could respond “yes” or “no”.

#### Interpersonal Factors

Interpersonal factors included emotional support, relationship with husband, relationship with mother, and relationship with mother-in-law. Emotional support was assessed by asking whether or not the respondent could receive emotional support and concern from family members or friends during pregnancy (no/yes). Relationship with husband, relationship with mother, and relationship with mother-in-law were assessed by asking whether these relationships were harmonious (harmonious/not harmonious).

#### Worries and Impacts on Daily Life

Worries and impacts on daily life included worries about fetal sex, worries about appearance, and changes to daily life. Respondents were asked whether or not they were worried about the sex of the fetus or their physical appearance due to pregnancy (no/yes) and whether or not pregnancy had impacted their daily life (no/yes).

#### Measurement of Perceived Stress

The Perceived Stress Scale (PSS), which was used to measure the perception of stress (22), consists of 10 items involving questions about one’s feelings and thoughts in the past month. Each item is answered on a five-point Likert scale ranging from 0 (never) to 4 (very often). The overall score ranges from 0 to 40, with higher scores indicating higher levels of perceived stress. The Cronbach’s alpha coefficient for this scale in this study was 0.810.

#### Measurement of Resilience

The 14-item Ego Resilience Scale was applied to assess resilience (23). Each item was rated on a seven-point scale ranging from 1 (strongly disagree) to 7 (strongly agree). Higher scores represent better levels of resilience. This scale has been widely used in previous studies in China with satisfactory reliability (24). The Cronbach’s alpha coefficient for this scale in this study was 0.957.

#### Measurement of Prenatal Depression

Depression symptoms were measured using the 20-item Center for Epidemiologic Studies Depression Scale (CES-D) (25), a questionnaire that has commonly been used to measure depression in China. Pregnant women reported their feelings and experiences with regard to depression and behaviors to cope with it “during the past few weeks” at any stage during their pregnancy. Each item was scored using a range from 1 (strongly disagree) to 7 (strongly agree). Summed CES-D scores of 16 or higher indicate that individuals are suffering from depression. The Cronbach’s alpha coefficient for this scale in this study was 0.855.

### Data Analysis

The distribution of CES-D scores, which was organized into categorical variables, was evaluated using the chi-squared test. The kappa test was used to evaluate the predictive variables, and the results of all kappa values were under 0.50, which implied no agreement, and the variables were not adjusted in the multivariate analysis. Multivariate logistic regression analysis was performed to determine risk factors associated with prenatal depression. The statistical analyses were conducted using SPSS statistical software for Windows version 17.0 (SPSS, Inc., Chicago, IL). A two-tailed *P*-value of less than 0.05 was considered to be statistically significant.

## Results

### Descriptions of Demographic Characteristics of Pregnant Women

The demographic characteristics of the participants are shown in **Table 1**. A total of 605 pregnant women at 12 to 40 weeks of gestation participated in the study. There were no significant differences in the demographic characteristics (age, chronic disease status, and weeks of gestation) between the included 605 pregnant women and the 195 women who were excluded from the study (**Table 2**). The age of the participants ranged from 19 to 42 years old, with a mean age of 30 years. Most of the women (67.1%; 406) were at 28 or more weeks of gestation. Pregnant women who did not have harmonious relationships with their husbands, mothers, and mothers-in-law comprised 7.4% (45), 5.5% (33), and 19.5% (118) of the population, respectively. As shown in **Table 1**, scores of 26 and 80 were cut-off values for perceived stress and resilience, respectively. Thus, 52.9% (320) of participants experienced high levels of stress and 49.4% (299) reported high levels of resilience.

**Table 1:** The demographic characteristics of the participates and univariate analysis of factors related to prenatal depression of pregnant women (N=605).

Variables	Number(%)	Non-Prenatal depression	Prenatal depression (CES-D≥16)	*P*
**Age (years)**				
< 30	289(47.77)	204	85	0.857
30-34	226(37.35)	163	63	
≥ 35	90(14.88)	66	24	
**Education**				
Junior college and below	298(49.3)	207	91	0.149
Bachelor's degree and above	307(50.7)	226	81	
**Monthly income (yuan)**				
≤ 3000	89(14.7)	49	40	0.001**
3001-5000	260(43.0)	189	71	
> 5000	256(42.3)	195	61	
**Weeks of gestation**				
< 28	199(32.9)	129	70	0.007**
≥ 28	406(67.1)	304	102	
**Chronic diseases**				
No	560(92.6)	407	153	0.028*
Yes	45(7.4)	26	19	
**Emotional support**				
Yes	485(80.2)	369	116	<0.001**
No	120(19.8)	64	56	
**Relationship with husband**				
Harmonious	560(92.6)	408	152	0.013*
Not harmonious	45(7.4)	25	20	
**Relationship with mother-in-law**				
Harmonious	487(80.5)	366	121	0.001**
Not harmonious	118(19.5)	67	51	
**Relationship with mother**				
Harmonious	572(94.5)	422	150	<0.001**
Not harmonious	33(5.5)	11	22	
**Worries about fetal sex**				
No	522(86.3)	389	133	<0.001**
Yes	83(13.7)	44	39	
**Worries about appearance**				
No	265(43.8)	213	52	<0.001**
Yes	340(56.2)	220	120	
**Impacts on daily life**				
No	279(46.1)	222	57	<0.001**
Yes	326(53.9)	211	115	
***Perceived stress***				
≤ 26	285(47.1)	244	41	<0.001**
> 26	320(52.9)	189	131	
***Resilience***				
≤ 80	306(50.6)	177	129	<0.001**
> 80	299(49.4)	256	43	

^*^Significant at the 0.05 level (two-tailed); ^**^ Significant at the 0.01 level (two-tailed)

**Table 2:** The characteristics of the participates and the excluded.

Variables	Participants（n=605）	Excluded （n=195）	χ^2^	*P*
**Age (years)**			1.253	0.535
<30	289	93		
30-34	226	67		
≥35	90	35		
**Weeks of gestation (week)**			0.304	0.323
<28	199	60		
≥28	406	135		
**Chronic diseases**			1.569	0.136
No	560	175		
Yes	45	20		

### Descriptions of Prenatal Depression in Pregnant Women

The prevalence of prenatal depression among Chinese pregnant women was 28.4%. The distribution of prenatal depression is presented in **Table 1**. Participants with a monthly income of less than 3,000 yuan had a higher prevalence of prenatal depression than women in other income groups (*P* < 0.01). Women whose gestation was less than 28 weeks had a higher prevalence of depression than women in later stages of pregnancy (*P* < 0.01), as did participants who suffered from chronic diseases (*P* < 0.05). Participants who could not receive emotional support from families and friends had a higher prevalence of depression than those who were able to receive support (*P* < 0.01). Participants who enjoyed harmonious relationships with their husbands, mothers, and mothers-in-law reported lower rates of prenatal depression than those who had poor relationships (*P* < 0.05). Additionally, participants who worried about their appearance, fetal sex, and pregnancy’s impacts on daily life had a higher prevalence of prenatal depression than those who did worry about such things (*P* < 0.01). Finally, women who had a higher level of perceived stress and a lower level of resilience had a higher rate of prenatal depression (*P* < 0.01). The Reliability Analysis of the Scales is provided in **Table 3**.

**Table 3:** The reliability analysis of the scales.

The instruments/questions		Number of items	Domains/ Sum score	Reliability- Cronbach's alpha coefficient
**Perceived stress**	The Perceived Stress Scale (PSS)	10	Sum score	0.810
**Resilience**	EGO Resilience Scale	14	Sum score	0.957
**Prenatal depression**	Center for Epidemiologic Studies Depression Scale (CES-D)	20	Sum score	0.855

### Predictors of Prenatal Depression

The results of multivariate logistic regression analyses of factors associated with prenatal depression are listed in **Table 4**. After adjusting for age and education, compared with a monthly income of less than 3,000 yuan, monthly incomes of more than 5,000 yuan (odds ratio [OR] 0.408, 95% confidence interval [CI] 0.217–0.768) and 3,000–5,000 yuan (OR 0.467, 95% CI 0.241–0.903) decreased the chance of suffering from prenatal depression. Worrying about appearance (OR 1.666, 95% CI 1.043–2.661) and higher levels of perceived stress (OR 1.156, 95% CI 1.104–1.211) increased the likelihood of prenatal depression. Pregnant women who had harmonious relationships with their own mothers (OR 0.287, 95% CI 0.103–0.796) and higher levels of resilience (OR 0.935, 95% CI 0.918–0.953) were less likely to experience prenatal depression.

**Table 4:** The multivariate logistic regression analysis for exploring factors of prenatal depression.

	OR	95%*CI*
**Monthly income**		
≤3000 yuan as the reference group		
3000-5000	0.467	0.241~0.903
>5000	0.408	0.217~0.768
**Weeks of gestation**	0.976	0.948~1.005
**Chronic disease**		
No as the reference group	1.238	0.560~2.735
Yes		
**Emotional support**		
No as the reference group		
Yes	0.638	0.361~1.125
**Relationship with husband**		
Harmonious as the reference group		
Not harmonious	1.668	0.673~4.135
**Relationship with mother in law**		
Harmonious as the reference group		
Not harmonious	1.073	0.597~1.930
**Relationship with mother**		
Not harmonious as the reference group		
Harmonious	0.287	0.103~0.796
**Worries about fetal sex**		
No as the reference group	1.429	0.770~2.654
Yes		
**Worries about appearance**		
No as the reference group	1.666	1.043~2.661
Yes		
**Impacts on daily life**		
No as the reference group	1.425	0.906~2.241
Yes		
**Perceived stress**	1.156	1.104~1.211
**Resilience**	0.935	0.918~0.953

-2Log L:524.458

OR, odds ratio; 95% CI, 95% confidence interval.

Age and education were fixed in the model.

## Discussion

In this study, the prevalence of prenatal depression in women in the middle and late stages of pregnancy in China was 28.4%, which was higher than the prevalances in high-income countries such as the United States (15%), Australia (7%), Hong Kong (4.4%), and Finland (7.7%), as well as low-income countries such as Pakistan (64.6%), Bangladesh (18%), Nigeria (24.5%), and Ethiopia (24.94%) (26). However, the prevalence we observed was lower than that reported by a study in India (36.7%) (26). In mainland China, depression is often overlooked and untreated. However, the mental health of pregnant women should be emphasized, since it is closely associated with not only the health of women themselves but also the growth and development of the fetus (21, 27). The present findings show that a significant proportion of Chinese women are vulnerable to the negative impacts of pregnancy, which can lead to the development of prenatal depression. The lack of attention to the mental health of pregnant women results in a reduction in the quality of prenatal and postnatal care and a higher prevalence of maternal and fetal complications (21, 27). In Chinese society, married women account for a significantly high proportion of pregnant women with prenatal depression, thus, this study was conducted among married women.

In this study, prenatal depression was not only associated with worries about appearance and perceived stress but was strongly linked to harmonious relationships with mothers. Furthermore, resilience played a positive role in attenuating depression, as observed in previous studies that showed that resilience could mitigate adverse childhood experiences that might lead to prenatal depression (28). Also, in this study, demographic characteristics, such as low income, accounted for some of the risk for prenatal depression, which was in agreement with previous studies (29). Women with lower incomes encounter considerable economic challenges and financial hardships, which increases the likelihood of prenatal depression.

Prenatal depression was best predicted by harmonious relationships with mothers. Among pregnant women, good relationships with their own mothers were negatively associated with prenatal depression, implying that a harmonious relationship between a pregnant woman and her mother could act as a protective factor to reduce the negative influences of pregnancy and mitigate the development of depression. In fact, the better a woman’s relationship with her mother, the lower levels of depression she experienced. Pregnant women can also receive social support from their mothers and take advantage of available resources to combat the detrimental impacts of stress and attenuate symptoms of depression. Our findings are in agreement with previous studies indicating that support from mothers could decrease the risks of mental and physical health problems among pregnant women (30).

Worries about appearance were positively associated with prenatal depression. Women experience physical changes and hormonal fluctuations (31) during pregnancy, which could result in considerable changes in their physical appearance, and, subsequently, their mental health may be severely affected, increasing the appearance of symptoms of depression. Likewise, the extent of perceived stress was a strong predictor of depression and has tremendous detrimental effects on the mental health of pregnant women. Pregnancy is a major transition that imposes numerous stresses on women, adding to the risk of depression during the perinatal period. Previous international studies revealed that most pregnant women reported perceived stress during pregnancy (32). Comprehensive stress management should be considered a priority in order to relieve depression and improve overall well-being for pregnant women.

Notably, in this study, resilience was strongly and negatively associated with prenatal depression, serving to attenuate the deleterious effects of pregnancy on depression, which was in agreement with previous studies that found that resilience was negatively linked to prenatal depression (33). Resilience is a positive psychological characteristic that helps pregnant women appropriately cope with stress and adversity and take advantage of available resources to deal with pregnancy-related stress and recover from challenges. Resilience can be protective against depression: it facilitates an individual’s ability to rationally combat the harmful effects of psychological challenges and enhances her ability to maintain mental well-being. Prenatal depression can be mitigated by the positive psychological trait of resilience, which was confirmed by a previous study (20). Pregnant women with more resilience might recover more quickly from difficulties by accessing positive resources more readily. In my study, we concluded that resilience was acted as not only a stable state-like positive psychological capability, which could be changed and improved, but also as a personality trait. This was in agreement with the recent study of measuring resilience by generalizability theory in breast cancer (34). Thus, counseling and psychotherapy initiatives such as stress management and resilience-enhancement training should be provided for women to reduce the negative effects of pregnancy on the prevalence of prenatal depression.

Early detection of risk factors for prenatal depression among women during pregnancy is important in order to reduce the risk of developing depression. Worries about appearance and perceived stress were the strongest risk factors for prenatal depression. Interpersonal factors, such as harmonious relationships with mothers, and resilience, as a protective factor against depression, played important positive roles in relieving the detrimental effects of pregnancy on prenatal depression. It is of vital importance for pregnant women to improve resilience and build harmonious relationships to help them adjust to additional stress and worries, access positive resources, and avoid prenatal depression. This study will help provide insight into the identification of high-risk pregnant women with emotional distress, give evidence for clinical interventions to improve mental health for pregnant women, and establish holistic and humanized prenatal care policies.

This study has several strengths. First, the large sample size allowed this study to explore the relationships between prenatal depression and interpersonal factors, worries and impacts on daily life, perceived stress, and resilience among Chinese pregnant women. Second, face-to-face interviews and smartphone questionnaires provided a high response rate for this study and guaranteed the accuracy of the information. However, due to the cross-sectional design of this study, conclusions about causality between prenatal depression and related factors could not be derived. Thus, findings from the present study need to be confirmed in future prospective studies. Finally, this research was conducted only in the married pregnant women and urban areas in mainland China, which may not allow for the generalizability of our results to other populations of the pregnant women.

## Ethics Statement

The research was conducted in accordance with the Declaration of Helsinki, as revised in 1989, and the protocol was approved by the Ethics Committee of China Medical University (CMU2018-033). All subjects gave their online informed consent for inclusion before participating in the study.

## Author Contributions

LZ contributed to the design of the study, acquisition of data, and revision of the manuscript. JZ critically reviewed the manuscript and provided English edits. WZ and CC contributed to the acquisition of data and revision of the manuscript. FY, RM, and YJ contributed to the acquisition and interpretation of data. XY conceived and designed the study. All authors read and approved the final manuscript.

## Conflict of Interest

The authors declare that the research was conducted in the absence of any commercial or financial relationships that could be construed as potential conflict of interest.
